# Combined protocol for severe and moderate acute malnutrition in emergencies: Stakeholders perspectives in four countries

**DOI:** 10.1111/mcn.12920

**Published:** 2019-11-26

**Authors:** Sarah L. Dalglish, Mamoudou Seni Badou, Amin Sirat, Omar Abdullahi, Mena Fundi Eso Adalbert, Marie Biotteau, Amelia Goldsmith, Naoko Kozuki

**Affiliations:** ^1^ Johns Hopkins Bloomberg School of Public Health Johns Hopkins University Baltimore Maryland; ^2^ Nutrition Department International Rescue Committee Niamey Niger; ^3^ Nutrition Department International Rescue Committee Maiduguri Nigeria; ^4^ Nutrition Department International Rescue Committee Mogadishu Somalia; ^5^ Nutrition Department International Rescue Committee Juba South Sudan; ^6^ Nutrition Department International Rescue Committee Bamako Mali; ^7^ Nutrition Department International Rescue Committee Washington, DC USA

**Keywords:** assessment of nutritional status, child nutrition, health policy, international child health nutrition, malnutrition, policy making

## Abstract

Each year, acute malnutrition affects an estimated 52 million children under 5 years of age. Current global treatment protocols divide treatment of severe acute malnutrition (SAM) and moderate acute malnutrition (MAM) despite malnutrition being a spectrum disease. A proposed Combined Protocol provides for (a) treatment of MAM and SAM at the same location; (b) diagnosis using middle‐upper‐arm circumference (MUAC) and oedema only; (c) treatment using a single product, ready‐to‐use‐therapeutic food (RUTF), and (d) a simplified dosage schedule for RUTF. This study examines stakeholders' knowledge of and opinions on the Combined Protocol in Niger, Nigeria, Somalia, and South Sudan. Data collection included a document review followed by in‐depth interviews with 50 respondents from government, implementing partners, and multilateral agencies, plus 11 global and regional stakeholders. Data were analysed iteratively using thematic content analysis. We find that acute malnutrition protocols in these countries have not been substantially modified to include components of the Combined Protocol, although aspects were accepted for use in emergencies. Respondents generally agreed that MAM and SAM treatment should be provided in the same location, however they said MUAC and oedema‐only diagnosis, although more field‐ready than other diagnostic measures, did not necessarily catch all malnourished children and may not be appropriate for “tall and slim” morphologies. Similarly, using only RUTF presented inherent logistical advantages, but respondents worried about pipeline issues. Respondents did not express strong opinions about simplified dosage schedules. Stakeholders interviewed indicated more evidence is needed on the operational implications and effectiveness of the Combined Protocol in different contexts.

**Key Messages**

- Acute malnutrition is a spectrum condition affecting over 50 million children underage 5 each year
- A proposed Combined Protocol to treat acute malnutrition provides for streamlined management of both severe and moderate forms in emergency settings
- Stakeholders in Niger, Nigeria, Somalia and South Sudan were stronglysupportive of providing treatment for both forms of malnutrition at the same location
- However stakeholders raised concerns around the proposed diagnostic criterion prioritizing mid‐upper arm circumference over weight‐for‐height z‐score and supply pipeline issues potentially affecting availability of ready‐to‐use therapeutic foods
- More research is needed on the clinical efficacy and operational implications of the Combined Protocol

## 1 INTRODUCTION

Each year, an estimated 52 million children under 5 years of age suffer from acute malnutrition (WHO, & World Bank Group, 2019). At least 500,000 children die each year from severe acute malnutrition (SAM; Black et al., 2013); these children also have weakened immunity and are susceptible to long‐term developmental delays and chronic disease later in life (Black et al., 2008; Lelijveld et al., 2016). Children suffering from moderate acute malnutrition (MAM) are also at higher risk of death compared with those who are not acutely malnourished (Chang et al., 2013), and moderate malnutrition can create lifelong problems due to cognitive development, and reduce lifelong earnings (Black et al., 2008). Food insecurity and hunger caused by ongoing and new conflicts and increasingly by climate change and migration mean that child malnutrition will remain an issue of the utmost concern.

**Figure 1:**
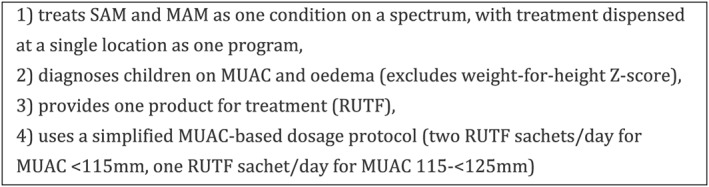
Key components of the Combined Protocol for treatment of MAM (moderate acute malnutrition) and SAM (severe acute malnutrition). For more information, see the trial protocol (Bailey J, Lelijveld N, Marron B, Onyoo P, Ho LS, Manary M, et al. Combined Protocol for Acute Malnutrition Study in rural South Sudan and urban Kenya: study protocol for a randomized controlled trial. Trials. 2018; 19(1): 251). MUAC, middle‐upper‐arm circumference; RUTF, ready‐to‐use therapeutic food

Global treatment protocols, adapted by most countries for use at national level, call for MAM and SAM to be managed separately, despite malnutrition being a spectrum disease. SAM is treated with ready‐to‐use therapeutic food (RUTF) in Outpatient Therapeutic Programs (OTPs), supplied and led by UNICEF. In the absence of codified global guidelines, MAM is often treated with ready‐to‐use supplementary food (RUSF) or corn soy blend++ in Supplementary Feeding Programs (SFPs), supplied and led by the World Food Programme (WFP). The International Rescue Committee (IRC) alongside other global actors has been researching the use of a Combined Protocol to manage both conditions, with diagnostic criteria adapted for emergency settings (only middle‐upper‐arm circumference, MUAC, and oedema instead of both MUAC and weight‐for‐height *z*‐score, WHZ, together with oedema) as well as a simplified dosage schedule. These approaches are meant to provide harmonized technical guidance, cost‐efficient supply management, and optimal care for the greatest number of children and families under challenging circumstances. This protocol was tested by the IRC in a randomized controlled trial known as the Combined Protocol for Acute Malnutrition (ComPAS) trial, with results pending (Figure 1; Bailey et al., 2016; Bailey et al., 2018).

Policy discussions about combining, simplifying, and/or improving current malnutrition treatment protocols have advanced among technical and financial partners at global and regional level, particularly in West Africa and to a lesser extent in East Africa. Versions of these approaches have found traction in conflict‐affected and/or food‐insecure settings where logistical constraints make delivery of the typical MAM/SAM protocols difficult if not impossible (Hanson, 2017; Woodhead, Rio, & Zagre, 2019). However, it remains unclear the extent to which these protocols are deemed acceptable and appropriate in countries where their use is currently being proposed. It is important to understand factors that influence national stakeholders' consideration and adoption of novel malnutrition protocols in emergency settings.

This study draws on four case studies to analyse national policy discussions and decision‐making around acute malnutrition policies in Niger, Nigeria, Somalia, and South Sudan. In each of these countries, rates of child malnutrition have reached emergency levels due to protracted political crises (Somalia and South Sudan) or more localized conflicts (Niger and Nigeria), often exacerbated by seasonal droughts and the effects of climate change. The objective of this article is to examine the perspectives of country‐level stakeholders with respect to proposed modifications to current MAM and SAM treatment protocols under the Combined Protocol in emergency settings.

## 2 METHODS

Case study methodology is used to reconstruct phenomena to reveal underlying processes and is thus well‐suited to policy analyses (Yin, 1994). We use case studies to understand stakeholders' knowledge and opinions around the four main components of the Combined Protocol and its potential use in emergency settings.

Data collection (March–July 2018) followed standardized guidance and included a desk review of relevant policy and program documents including national policies and guidelines on malnutrition, Ministry of Health (MOH) strategic plans and memos, implementation tools, and academic and research reports. Data collection took place mainly in capital cities (Niamey, Mogadishu, and Juba), except in Nigeria where it took place in Maiduguri, Borno State (phone interviews were conducted with Abuja‐based respondents). Semistructured interviews were conducted in countries (*n* = 46 interviews, *n* = 50 interviewees) and with stakeholders at global/regional level (*n* = 8 interviews, *n* = 11 interviewees; Table 1). Respondents included personnel of the MOH, UNICEF, WFP, and various NGOs in most countries. All respondents were asked about policy discussions at the national level including key actors and recent events; specific barriers or hesitations about modifying protocols; operational, financial, and practical considerations; and the importance of scientific evidence and global guidance. Most interviews took place in person, although some took place remotely via Skype or phone. Interviews lasted an average of 54 min (range: 33–82 min) and were conducted in English for stakeholders in Nigeria, Somalia, and South Sudan and in French in Niger and with some regional/global respondents. A small number of respondents requested not to be recorded; notes were taken for these interviews. Recorded interviews were transcribed verbatim then verified and completed by interviewers.

**Table 1:** Primary data collection

	Number of interviews	Number of interviewees	Number of documents reviewed
Niger	13	15	12
Nigeria	11	11	16
Somalia	12	12	12
South Sudan	10	12	11
Global/regional level	8	11	13
TOTAL	54	61	54

Data analysis was iterative and concurrent with data collection, beginning with regular debriefing discussions between lead data collectors to discuss emerging themes and adjust interview questions, combining analytical strands from the document review and interviews. Data from documents were abstracted into a standardized coding form to systematically track key themes, allowing for comparison across cases of theoretical and contextual categories (Maxwell, 2005). In‐country data collection concluded with debriefing sessions to share initial impressions with and seek any clarification from IRC's country nutrition program focal points. Subsequently, interview transcripts were analysed using thematic coding, with categories developed *a priori* based on the theoretical literature and research questions; emergent codes were also developed after testing the coding structure on a first set of interviews. Interviews were coded using NVivo software (Version 11); the final codebook included codes on the content of current malnutrition policy and protocols; policy processes in countries; policy interactions at country, regional, and global level; national context; and the origins, rationale, and arguments for or against combined/simplified protocols. A synthesis report was reviewed by in‐country IRC staff in all countries for accuracy of facts and interpretation, resulting in minor adjustments.

Ethical clearance was provided by the relevant bodies in all four countries and by the IRC Institutional Review Board.

## 3 RESULTS

Acute malnutrition protocols in all countries were based on global (World Health Organization, WHO) standards, although not all were current with the latest guideline updates (WHO, 2012). In Niger and Somalia, national guidelines are called Integrated Management of Acute Malnutrition, whereas in Nigeria and South Sudan these are called Community Management of Acute Malnutrition (CMAM). Respondents in all countries frequently mentioned that national guidelines were adapted in their essentials from the “global” (WHO) guidelines. Nonetheless, specific aspects of treatment protocols varied slightly by country (Table 2). Differences were particularly notable for MAM treatment, likely due to a lack of global guidance. In Somalia and South Sudan, children received supplementary feeding in Targeted Supplementary Feeding Programs (TSFPs); however, treatment for MAM was virtually absent in Nigeria, both from the protocol and in the field (in Borno state and nationally). Treatment for MAM was included in Niger's protocol; however, RUSF or other supplementary foods were available only in insecure regions and/or those with very high global acute malnutrition rates. Guidelines varied in terms of preference for MUAC over WHZ as screening and discharge criteria, although all had incorporated MUAC in some way. Modes of dispensing treatment also varied, although in most cases MAM and SAM care were not provided in the same place on the same day. In all countries, national guidelines were meant to be applied nationwide including in emergency settings. Next, we examine stakeholders' views on the four proposed components of the combined protocol, as laid out in Figure 1.

**Table 2:** Summary overview of national policies*

	Niger	Nigeria	Somalia	South Sudan
Name of acute malnutrition protocol	“National Protocol for Integrated Management of Acute Malnutrition” (2016)	“National Guidelines for Community Management of Acute Malnutrition” (2011)	“Somali Guidelines for Integrated Management of Acute Malnutrition” (2018)	Community Management of Acute Malnutrition (2017)
Location of treatment	SAM children treated at OTP (in health centre) or SC (in a hospital). MAM children may be referred to a health centre, but little RUSF is currently available. MAM and uncomplicated SAM cases are seen at the same health centre on different days.	SAM children are treated at OTP/SC (usually inside a health facility). MAM children may be referred to NGOs for local counselling/IYCF programs, if they exist.	SAM children at treated at OTP/SC, with MAM children treated at TSFP centres. The country is moving to ensure SCs, OTPs and TSFPs are henceforth all located together, ideally at health facilities, under the “Rationalization Plan.”	SAM children at treated at OTP/SC, with MAM children treated at TSFP centres. Sometimes these are at the same physical location but operate on different days. Discussion about moving OTPs and TSFPs to the same location.
Screening/admission/discharge with MUAC or WHZ	**SAM (SC)**: admission on either MUAC <11.5 ***OR*** WHZ < −3.0 ***OR*** bilateral oedema + , ++ (all with oedema +++, poor appetite or complications). **SAM (OTP):** admission on either MUAC <11.5 ***OR*** WHZ < −3.0 ***OR*** bilateral pitting oedema + , ++ (all without complications) and all with moderate or good appetite. **MAM (TSFP)**: admission on either MUAC ≥ 11.5 and <12.5 ***OR*** WHZ ≥ −3.0 and <−2.0. Discharge on ***BOTH*** MUAC and WHZ and all during two consecutive weeks or no oedema during 14 days MUAC and oedema−only is widely used for admissions in mass screenings & in emergency situations.	**SAM (SC)**: admission on either MUAC <11.5 ***AND/OR*** WHZ < −3.0 ***OR*** bilateral oedema +++ (or medical complications or kwashiorkor). **SAM (OTP):** admission on either MUAC <11.5 ***OR*** WHZ < −3.0 “if used” ***OR*** bilateral pitting oedema + , ++ (all without complications, and with moderate or good appetite. **MAM (TSFP)**: admission on either MUAC ≥11.5 and <12.5 ***OR*** WHZ ≥ −3.0 and <−2.0. Discharge on MUAC ***OR*** WHZ (“if used”) ***AND*** no oedema during 14 days MUAC and oedema−only is recommended at facility and community level (WHZ is “time consuming”), as well as for mass screenings & in emergency situations.	**SAM (SC)**: admission if MUAC <11.5 (OTP) ***OR*** WHZ (<−3) ***OR*** bilateral oedema + , ++ (all with oedema +++, poor appetite or complications). **SAM (OTP)**: admission on MUAC <11.5 ***OR*** WHZ < −3.0 ***OR*** bilateral pitting oedema +, ++ (without complications). **MAM (TSFP)**: admission on either MUAC ≥ 11.5 and <12.5 ***OR*** WHZ ≥ −3.0 and <−2.0. Discharge criteria are unclear as guidelines are under revisions. Under current guidelines, the discharge criteria for both OTP/TSFP is >12.5 or>−2. Where there is TSFP site, the discharge criteria for OTP is >11.5 but where there is no TSFP, the discharge is >12.5. MUAC used commonly in emergency situations.	**SAM (SC):** admission if MUAC <11.5 (OTP) ***OR*** WHZ (<−3) ***OR*** bilateral pitting oedema+++ (all with complications). **SAM (OTP)**: admission on MUAC < 11.5 ***OR*** WHZ < −3.0 ***OR*** bilateral pitting oedema + and ++ (all without complications). **MAM (TSFP):** admission on either MUAC ≥ 11.5 and <12.5 ***OR*** WHZ ≥ −3.0 and <−2.0. Discharge on the **same criterion** (MUAC of WHZ) used for admission and no oedema during two consecutive weeks If no TSFP, refer to “expanded criteria” for discharge. MUAC used commonly in emergency situations.
Therapeutic products in use	SAM: RUTF (Plumpy'Nut) MAM: RUSF, CSB++ (or nothing)	SAM: RUTF (Plumpy'Nut) MAM: Super Cereal Plus (or nothing)	SAM: RUTF (Plumpy'Nut) MAM: RUSF (Plumpy'Sup)	SAM: RUTF (Plumpy'Nut) MAM: RUSF (Plumpy'Sup, CSB++)—although with frequent stock−outs
Dosage	RUTF by weight for SAM. One sachet of RUSF per day for MAM (not usually available) Local products indicated in case of stock−out.	RUTF by weight for SAM, No mention of RUSF ration for MAM in current protocol	RUTF by weight for SAM, One sachet/day RUSF for MAM.	RUTF by weight for SAM One sachet/day RUSF for MAM.
Exceptions for “emergencies”	A proposed annex to the current protocol gives a table for reduced dosage (see Annex 5) in case of supply shortages and other emergencies, however it has not been formally adopted.	None officially stated.	Exceptions to the national protocol granted to use a single product in case of stockouts, or if there is no OTP/TSFP in a certain area. The process appears to be informal. Rapid Response Mechanism teams also use a simplified dosage scheme (two RUTF/child).	Expanded Criteria is part of the CMAM guidelines, to be used in emergencies as a “stopgap” measure (Annex 4). Permission is granted via a formal process involving the Nutrition Cluster, UNICEF and WFP.

*Note*. There may be deviations from documented guidelines in practice.

Abbreviations: CMAM, Community Management of Acute Malnutrition; CSB++, corn soy blend++; MAM, moderate acute malnutrition; MUAC, middle‐upper‐arm circumference; OTP, Outpatient Therapeutic Program; RUSF, ready‐to‐use supplementary food; RUTF, ready‐to‐use‐therapeutic food; SAM, severe acute malnutrition; TSFP, Targeted Supplementary Feeding Programs; WHZ, weight‐for‐height z‐score.

### 3.1 Agreement that MAM & SAM should be treated at the same location

Stakeholders displayed strong agreement about the desirability of providing SAM and MAM treatment at the same location in Nigeria, Somalia, and South Sudan. In Nigeria, where MAM treatment is not prioritized either in national guidance or in the field, stakeholders were nonetheless enthusiastic about integrating treatment in the same location:

“Basically if you ask me about the effectiveness having both MAM and SAM treatment at the same place I can say you are hundred percent very relevant.” ( National NGO, Nigeria)

In Somalia and South Sudan, there was also strong acceptance of providing MAM and SAM treatment in the same place, and respondents frequently characterized the main problem with malnutrition care as being the fact that OTPs and TSFPs were often not placed in the same location:

“If you see a mom with a SAM with complications child at your [stabilization center] and … you say to the mom, ‘I will refer you in a two kilometers or three kilometers away to the OTP program,' she will face some obstacles, starting from the transportation.” ( National NGO, Somalia)

In Somalia, recent efforts to improve acute malnutrition services have included implementation of a three‐phase “Rationalization Plan,” to correct the poor coordination observed during the 2011 famine. In a first phase, stakeholders documented caseloads and mapped operations of implementing partners to reduce redundancies and provide clear mandates to each. The Somalia Nutrition Cluster led the process of geotagging every OTP and TSFP in country with the goal of integrating them. At the time of this study, this process was complete in Somaliland, about halfway finished in Puntland, and furthest behind in the Central South region, where insecurity is greatest (multilateral agency, Somalia). Starting March 2018, the country's latest Rationalization Plan required partners working in certain areas to provide both programs at the same site and/or coordinate with the other actors:

“We have committed wherever there's TSFP or OTP, the other partner should provide the other component to treat.” ( Government official, Somalia)

In South Sudan, the ideal of having treatment for both conditions take place at the same location also holds and is advocated by MOH, UNICEF, WFP, and the Nutrition Cluster:

“Actually our desire is to move towards having the same NGO implementing TSFP and OTP in the same location.” ( Multilateral agency, South Sudan)

Guidance in South Sudan provides for integration of OTPs and TSFPs, and, as in Somalia, the absence of one or the other of these facilities is viewed as a reason to use adaptations of the sort contained in the Combined Protocol.

Sometimes, treatment for MAM and uncomplicated SAM are provided at the same location but on different days (this was mentioned in Niger and South Sudan). Respondents in both countries mentioned the burden this created for families with children with both MAM and SAM:

“It's true, it's a bit onerous in the sense that a mom who has a SAM child and a MAM child, she has to come on both Monday and Tuesday.” ( National NGO, Niger)

However, in Niger, respondents from the government sector tended to defend the Nigerien system, which includes CRENI (*Centre de récupération et d'éducation nutritionnelle intensif*, or Stabilization Center (SC) in a hospital), CRENAS (*Centre de récupération nutritionnelle ambulatoire pour la malnutrition sévère*, or OTP for SAM in a health center), and CRENAM (*Centre de récupération nutritionnelle ambulatoire pour la malnutrition modérée*, or MAM treatment in a health center):

“The referral system, as the protocol has provided for, it's not complicated! All the actors are charged with carrying out the different processes. In my opinion, really, it's not complicated!” ( Government official, Niger)

### 3.2 Acceptability of MUAC for screening, but not always as the sole measure

In all countries, MUAC is widely used in mass screenings, for example, in camps for internally displaced persons and other insecure locations, and as part of Rapid Response Mechanisms. Pilot projects teaching mothers and other caregivers to screen their children using MUAC themselves (“Mother MUAC”) were also viewed positively in Niger, Nigeria, and Somalia. Nearly across the board, in all countries, respondents agreed that MUAC was a simple measure that was easy to use, a distinct advantage in emergencies:

“When we are dealing with emergencies such as the [internally displaced persons] and such a community ... the MUAC criteria is the best to use, because it is simple.” ( National NGO, Somalia)

“It's less time‐consuming, you don't have to spend a lot of time in those Al‐Shabaab controlled areas.” ( Multilateral agency, Somalia)

Specifically, MUAC was said to be faster, could be used by health workers with limited literacy and numeracy, could be performed by a single health worker, and was field‐ready (instead of requiring a battery‐powered scale, as for WHZ). A handful of respondents said that MUAC was a more sensitive measure compared with WHZ, and while others said it could miss a few outliers, this disadvantage could be offset by practical advantages:

“It's fine if we have a few false positives, let's [treat] them anyway.” ( Bilateral agency, South Sudan)

“I have no problem with screening with MUAC … if it means you are going to perhaps miss few children because of that not fitting the outliers versus missing a lot of children because you can't carry around a board and scale and do the calculation … to any degree of accuracy, then it's its fine with me.” (Multilateral agency, federal level, Nigeria)

Speaking about emergency situations, most people found the trade‐off between the perceived accuracy of WHZ and the ease of use of MUAC to be an acceptable one. MUAC's simplicity was often contrasted with the time‐consuming process of taking WHZ:

“You need to have a big resource to do *Z*‐score, to taking weight to taking height and to calculate because a lot of mothers are waiting … and it will make you kind of disappointing to your beneficiaries.” ( International NGO, Nigeria)

Indeed, WHZ is deprioritized in the Nigerian national protocol: it is described as “more time‐consuming” and cannot be used as an independent admission criterion at a health centre, whereas MUAC can be. WHZ was also deemed problematic in South Sudan, due to the low education level of health workers:

“Due to the low cadres we have in the field, the *Z*‐score becomes a bit too complicated for the community component.” ( International NGO, South Sudan)

Respondents in all countries mentioned that using MUAC instead of WHZ would reduce the burden on health workers, and free them up for other tasks, and agreed this was in principle a good thing:

“The essential thing is to lighten the task load.” (Government official, Niger)

However, there was far less agreement about MUAC's appropriateness as the sole criterion for diagnosis and discharge, particularly outside of emergency situations. A number of objections were raised. First, many respondents said that MUAC and WHZ were not interchangeable measures, although a smaller number were able to provide specific details about the differences between the two:

“I think you have seen a lot of publications going around, including recently by Mark Myatt and the [U.S. Centers for Disease Control]. I think the reality is that those two, MUAC and weight to height, do not measure the same thing.” ( Multilateral agency, South Sudan)

More commonly, a few respondents each in Somalia, South Sudan, and to a lesser extent Niger said MUAC was not appropriate for “naturally slender” body types found in these countries and/or among pastoral people (the issue of body type was not mentioned in Nigeria).

“When you go out into the regions, there are children who are naturally thin. Using the MUAC, in our society, here in the Sahel, MUAC only, well we, we haven't accepted it for the moment!” ( Government official, Niger)

In South Sudan, it was widely agreed by respondents that MUAC and oedema‐only admissions were inappropriate, given that children would be missed if WHZ were removed. There was strong agreement that South Sudanese anthropometry made MUAC unreliable, reportedly leading one proponent of MUAC and oedema‐only admissions to quit the recent CMAM guideline development process.

Strong objections to a MUAC and oedema‐only protocol were also raised in Niger along different lines: quality of care. In Niger, national policymakers perceived that they had been “lobbied” for MUAC and oedema‐only by stakeholders from international donors starting in 2014–15. Government officials opted to retain WHZ as one possible criteria for admission, and a necessary criterion for discharge. Respondents, particularly those in government, appeared annoyed when asked about a MUAC and oedema‐only protocol, asserting strongly that WHZ was essential because it is a superior measure for growth monitoring, and is already used throughout the health system for this purpose:

“You agree with me, a correct anthropometry requires weight‐for‐height.” ( Government official, Niger)

“It's true, when you have to measure weight, height, it requires … several people and it takes time, but it's not … wasted time!” ( Government official, Niger)

A number of non‐government respondents in Niger characterized government policymakers as being particularly conservative on this point, and recounted the difficulty of convincing the latter to include MUAC as one possible admission criterion (alongside WHZ):

“For [MUAC] to be an independent admission criteria, it required a memorandum from the Ministry … and it was a long battle for that to be accepted.” ( International NGO, Niger)

### 3.3 Reservations about the appropriateness of RUTF as the single product

Respondents expressed different opinions on whether RUTF and RUSF were interchangeable products from a clinical standpoint. The majority of respondents said they were very similar, but others said there were significant differences in terms of composition or nutritional content:

“Of course the contents are very different … We are talking about the micronutrients here.” ( Multilateral agency, South Sudan)

Respondents more frequently deemed it acceptable for MAM children to be treated with RUTF than for SAM children to be treated with RUSF, while acknowledging that RUSF was better than nothing, if RUTF were not available:

“Basically for RUTF we may use that to treat Moderate Acute Malnutrition but we are not ready to supply supplementary food to treat Severe Acute Malnutrition because the recipes or the ingredients are totally different.” ( International NGO, Nigeria)

“When we use RUSF to treat children with severe acute malnutrition, that is not the product meant to treat them, so in terms of quality of care, in terms of duration of treatment … it's just a stopgap measure.” ( Multilateral agency, South Sudan)

Generally speaking, if one product were to be used, or had to be used due to emergency conditions, a majority of respondents agreed that RUTF was superior to RUSF. Often, it seemed that respondents were open to the idea of a single product, as long as they could be assured this was clinically justifiable:

“If scientifically it can be proved that … they could get the product one product for the two—why not? That makes it easier.” ( Government official, Nigeria)

“If scientifically, with evidence, one product can serve for the two forms [of malnutrition], I think it would be a good thing … In my opinion, it would be a very good thing if solid scientific proof could be established.” ( Government official, Niger)

Many respondents acknowledged the benefits of using a single product in terms of simplicity and supply chain issues and therefore indirectly on staff time spent managing supplies:

“It will be easier for the management purpose and all the process of logistics and so on and so forth.” ( International NGO, South Sudan)

However, others warned of a potential problem with using RUTF and RUSF interchangeably given the current division of labour between UNICEF and WFP in estimating supply orders and monitoring use:

“UNICEF doesn't buy into this. For … all the supplies used … there should be a corresponding number of SAM children treated, you see? The same with … WFP and with MAM. WFP will only be interested in looking at the number of MAM cases that you treated.” ( International NGO, Somalia)

Comparing the two products from the standpoint of logistics and availability, the pipeline for RUTF was deemed as more reliable than the pipeline for RUSF in all countries. In Niger and Nigeria, this was obviously the case—in recent years, WFP funding for MAM treatment has been reduced, and little RUSF is available in either country. In Somalia and South Sudan, respondents said RUSF was available in some areas, but not others, because WFP is not allowed to subcontract monitoring in conflict areas (as UNICEF can)—although Rapid Response Mechanisms can and do bring RUSF with them. In contrast, although the supply of RUTF was frequently mentioned as costly, actual stock‐outs appeared rare.

Because responsibility for MAM and SAM is divided between WFP and UNICEF and funding and staffing levels are often contingent on the amount of RUSF and RUTF supplied, respectively, stakeholders voiced concerns about moving toward a single product. Protocols seen as threatening existing dispositions could cause strains at country level:

“At Somalia level … the Cluster came in very strongly and said ‘Let's not talk about the Combined Protocol. It will raise mandate issues, and we don't want to be part of that.'” ( International NGO, Somalia)

Global‐ and regional‐level respondents confirmed these issues; one said that “UNICEF and WFP kind of have a turf war going on,” since WFP has become increasingly involved in managing MAM (bilateral agency, global level). Another global respondent said:

“I feel that UNICEF and WFP at the global level, I still think, are tiptoeing around their own organizational mandates and challenges around anything to do with revisiting how this whole spectrum of acute malnutrition is dealt with.” ( Bilateral agency, global level)

### 3.4 Views on simplified dosage are pending, following decisions about previous components

Compared to the three other components of the Combined Protocol reviewed in this study, respondents had less well‐defined views regarding the use of the proposed simplified MUAC‐based dosage schedule (two RUTF sachets/day for MUAC < 115 mm, one RUTF sachet/day for MUAC 115–<125 mm). When asked, respondents tended not to provide strong arguments one way or the other, perhaps because the choice of dosage protocol would depend on a number of antecedents, specifically around the use of MUAC as a diagnostic criterion, and RUTF as the sole product. Although respondents were generally favourable toward proposals that increased simplicity and reduced the burden on health workers, it is difficult to say from our data how stakeholders view the proposed simplified dosage protocol or the notion of simplified dosage in general.

One related issue that was brought up only once (in Niger) but bears mentioning is the existence of conflicting paradigms around dosage schedules for acutely malnourished children. Specifically, one respondent (Multilateral agency, Niger) said that government policy actors in Niger and other West African countries were influenced by protocols calling for smaller doses of RUTF at earlier stages of recovery from SAM then increasing dosage as the child improves and requires greater caloric intake. This paradigm is reflected in the work of Michael Golden and Yvonne Grellety, who were funded by UNICEF to review guidelines in Eastern and Southern Africa (National NGO, Somalia). Country‐level policymakers who are familiar with these protocols may be less receptive to other protocols that are perceived as not following this paradigm. As the UN official in Niger said,

“There's this reticence … to completely embracing projects that don't have [the Golden protocol], [including] all the projects that talk about the [Combined Protocol]. Because people aren't necessarily convinced by the initial hypothesis.” ( Multilateral agency, Niger)

## 4 DISCUSSION

This study compared the views of malnutrition stakeholders in four countries with different types of emergencies on the Combined Protocol for treatment of MAM and SAM. National guidelines, which had either recently been updated or were currently being updated in all four countries, generally did not reflect the innovations proposed in the Combined Protocol, whether with respect to location of treatment, diagnostic criteria (MUAC and oedema only), treatment product, or dosage schedule. Respondents had different levels of knowledge about, and opinions regarding, the four main components of the Combined Protocol reviewed in this study. The first component, provision of treatment for MAM and SAM in the same physical location, was most well‐supported by respondents, who agreed this was logical and desirable. However, there were much stronger reservations about the acceptability of MUAC as a sole diagnostic measure (due to concerns about its appropriateness for Sahelian morphologies and as an indicator of growth monitoring) and about the use of RUTF for both MAM and SAM (due to concerns about nutritional content and potential mandate and funding issues between WFP and UNICEF). Regarding the simplified dosage protocol, respondents appeared to withhold judgment, as this would depend on decisions about the previous two components.

Flaws in current delivery systems of nutrition interventions during periods of food insecurity and emergency have long been recognized by stakeholders in the nutrition sphere (Collins, 2001; Woodhead et al., 2019). The Combined Protocol represents one of several proposed solutions to perceived difficulties in delivering needed care to acutely malnourished children in emergency situations. In March 2019, stakeholders at WHO, the Office of the United Nations High Commissioner for Refugees, UNICEF, and WFP met in Geneva to discuss and review simplified approaches for the treatment of child wasting (WHO, UNHCR, UNICEF,, & WFP, 2019). After more than a decade of scaling up current approaches, participants reviewed operational lessons, notably in the context of emergency situations and fragile health systems, focusing on issues of cost, health system infrastructure, national policy, coverage, and quality. The organizations represented agreed to pursue further investigation and documentation of simplified approaches “in exceptional circumstances, where warranted,” develop updated guidance on wasting in all settings (led by WHO), and present a global action plan on wasting by end 2019. As such, in addition to the ComPAS study in South Sudan and Kenya, a number of country‐level pilots and research projects on simplified are currently underway, notably in West and Central Africa (Woodhead et al., 2019).

More efficient and cost‐effective ways of preventing and treating SAM would be much welcomed by stakeholders at national and global levels, given the expense of this intervention, both in children's lives and in budgetary expenditures. A cost analysis of one large, well‐established community‐based program to treat SAM in Niger found an average cost per child of 149 euros (US$ 175 in today's currency), with cost breakdowns of US$ 83 for outpatient treatment costs and US$ 149 for inpatient treatment costs, respectively (Isanaka et al., 2017). Indeed, among 10 interventions recommended to improve maternal and child nutrition, SAM management was by far the most expensive, estimated at a total cost of $2.6 billion to achieve 90% coverage in the 34 highest‐burden countries (2010 international dollars; Bhutta et al., 2013). Yet, the same study also showed that effective management of MAM and SAM was among the most cost‐effective interventions per life saved, potentially saving up to 435,000 lives per year, when scaled up alongside the other nine interventions.

As a result of ongoing research, evidence is beginning to emerge that simplified approaches can provide safe, effective treatment in emergency settings. In Sierra Leone, a cluster‐randomized controlled trial used an integrated protocol with a single product (RUTF) and MUAC‐only admission (<12.5 cm) to test recovery and coverage rates (Maust et al., 2015). The trial protocol provided a decreased ration of RUTF when children reached MAM as measured by MUAC >11.4 and <12.5 cm and counselling messages delivered to parents by peers; the control arm used WHZ for admission and treated MAM with a fortified blended flour and SAM with RUTF. The results indicated greater coverage (number of children who received treatment/number of children eligible for treatment) in communities with integrated management (71 vs. 55%, *P* < .001), with comparable global acute malnutrition recovery rates [910 of 1,100 children (83%) with the integrated protocol, versus 682 of 857 (79%) with the standard]. In northern Burkina Faso, a CMAM program switched from proportional weight gain to MUAC ≥124 mm as the sole discharge criterion, and achieved a slightly higher recovery rate (Risk Ratio: 1.04, 95% CI [1.03, 1.05]) in a sample of 50,841 children (Isanaka et al., 2019). SAM children who were discharged in the second period using the MUAC‐based criterion also had shorter lengths of stay and fewer adverse program outcomes. Further evidence is awaited from the ComPAS trial (Bailey et al., 2018) and research by WFP, UNICEF, European Civil Protection and Humanitarian Aid Operations, and other partners in West Africa (Kaul, Husain, Tyrrell, Gaarder, & Jimenez, 2018).

There have been fewer trials of combined/simplified protocols in South Asia, although this region is home to the world's largest number of severely wasted children (up to 70% of the global burden) (Ahmed et al., 2014). Nonetheless a few country programs and projects have adopted some of the proposed modifications discussed here, albeit in nonemergency settings. In India, a CMAM program initiated by Médecins Sans Frontières in Bihar state that admitted children using MUAC < 115 mm for admission and MUAC ≥ 120 mm for discharge attained low mortality and high cure rates, in part by lowering the threshold for severity compared to WHZ < −3 *SD* (Burza et al., 2015). Pakistan's CMAM program has also achieved good recovery and survival rates using Lady Health Workers to screen and refer children based on MUAC < 115 mm (Aguayo et al., 2018). However, care should be taken in comparing these programs given that acute malnutrition in the settings studied in our analysis is driven by severe food insecurity (often prefamine warning levels), whereas the drivers of wasting may be different in South Asia.

Nonetheless, it is clear that more evidence is needed on specific aspects of combined/simplified protocols before national decision‐makers will substantially revise national protocols. For example, the hesitancy to adopt MUAC and oedema‐only diagnosis observed in our study is understandable given the lively and ongoing scientific debate on this topic (Briend et al., 2016; Grellety & Golden, 2016a). Compared with WHZ, there is broad agreement that MUAC constitutes the superior measure for use in community screenings, as well as in emergency situations, where obtaining WHZ is often infeasible. Médecins Sans Frontières has found success with MUAC in CMAM programming in emergency contexts, using admission thresholds varying from <115 to <125 mm, with outcomes for recovery, mortality, and default surpassing Sphere Minimum Standards in most cases (Hanson, 2017; Phelan et al., 2015). Although some analyses have suggested that up to three‐quarters of severely malnourished children are missed by MUAC <115 mm criteria (Grellety & Golden, 2016b), most proposed MUAC‐only protocols use a higher threshold than 115 mm. Interventions have sometimes used the sitting height to standing height ratio (or Cormic index) to correct of weight‐for‐height measurements for the body morphology of some ethnic groups (Roberfroid et al., 2015; Salama et al., 2001), but the issue of geographical variations body shape requires further study (Briend et al., 2016). Evolving understandings of the frequency of multiple anthropomorphic deficits and the overlap and dynamic interactions between wasting and stunting will also influence this debate (Myatt et al., 2018; Wells et al., 2019). Although it is beyond the scope of this article to adjudicate the debate around MUAC versus WHZ, greater clarity around the specific conditions under which MUAC is an appropriate diagnostic tool, emphasizing its practicality and ease in difficult field situations, will be needed to assuage stakeholders' observed misgivings.

Country‐level stakeholders also need more information about the appropriateness of RUTF as the single product to treat both MAM and SAM. Both products are lipid‐based nutrient pastes containing similar amounts of protein, although usually from (more expensive) animal sources for RUTF or from soy or whey for RUSF; amounts of vitamins and micronutrients also vary between the two products (Isanaka et al., 2010). RUSF was developed for treatment of MAM following the observed success of RUTF for treating SAM; however, RUSF is meant to supplement, not replace, other sources of nutrients. Yet the physiology of weight gain is essentially the same between MAM and SAM (Briend et al., 2015), suggesting potential exchangeability between products, although few studies exist to test this proposition. Although RUTF has been used successfully to treat large numbers of MAM children (Defourny, Seroux, Abdelkadar, & Harczi, 2007), one recent systematic review comparing various lipid‐based nutrient supplements for treatment of MAM found a higher recovery rate in children receiving RUTF compared to RUSF (RR 1.23, 95% CI [1.09, 1.38]; one study for RUTFs; RR 1.06, 95% CI [1.01, 1.11] for RUSFs; *P* = .02 for subgroup differences; Gera, Pena‐Rosas, Boy‐Mena, & Sachdev, 2017). An earlier study in Niger found a lower risk of wasting with RUSF compared with RUSF; however, this difference was attributed to factors related to a previous intervention (Isanaka et al., 2010). More research is needed about the relative efficacy, including weight gain but also micronutrient absorption and functional outcomes, of RUTF, RUSF, and their variants, as well as the cost effectiveness of the different options (Briend et al., 2015; Schoonees, Lombard, Musekiwa, Nel, & Volmink, 2019).

This study has some limitations. Not all interviewees were able to be reached in‐person in all study countries due to the short duration of visits for data collection. In Somalia in particular, the researcher's time in country was restricted due to security protocols, which also made it difficult to reach many potential respondents, although additional stakeholders were interviewed in Nairobi. We mitigated this limitation by conducting additional phone interviews and triangulating with documents, reaching saturation on most relevant points. However, given small sample sizes, it remains possible that some perspectives were not fully represented. Next, data were collected by a team of two researchers one in Niger and northeast Nigeria (SD) and the other in Somalia and South Sudan (NK). To ensure congruity, the two researchers held regular and frequent discussions, including debriefing during or shortly after field visits and throughout data analysis. Next, given that NK is an IRC employee, and SD an IRC consultant at the time of data collection, it is possible that some respondents may have tailored their answers on the Combined Protocol, which is often associated with IRC. We used triangulation between respondents, as well as probing on potentially controversial aspects of the protocol, to mitigate this risk. Finally, a handful of interviews at both country and regional level took place with two participants, rather than individually. Given that participants in these settings may have refrained from sharing negative views, particularly regarding their own organization, we interpreted these interviews as representing an institutional standpoint, rather than two individual actors.

Field‐level stakeholders are interested and aware of the potential benefits of simplified approaches to treating child wasting in emergency settings, including those included in the Combined Protocol. Further research on specific aspects of these protocols is needed to answer scientific and operational questions, as is already recognized by regional‐ and global‐level stakeholders, especially on issues where is a lack of consensus. Ongoing dialogue between these levels, and especially with national and MOH officials, will ensure planned research incorporates and answers field‐level concerns. At country level, stakeholders should be informed about ongoing research and be alert to ways to improve the effectiveness and cost efficiency of their own acute malnutrition programs, including as these may be affected by any global guideline revisions.

## CONFLICT OF INTEREST

All authors were employees of the IRC at the time of research, except SD and AG, who were also affiliated with IRC as either consultant or intern.

## CONTRIBUTIONS

SD and NK designed the study and conducted in‐country data collection, with support and input from SM, AS, OA, MFEA, and MB. AG conducted the desk review and document analysis with SD and NK. Data analysis was conducted by SD and NK, who also wrote the paper, with input from all authors. The final version was approved by all authors.
